# Supplementary material for: In Situ Preparation of Tannic Acid-Modified Poly(N-isopropylacrylamide) Hydrogel Coatings for Boosting Cell Response
Source: Pharmaceutics. 2024 Apr 13;16(4):538. doi: 10.3390/pharmaceutics16040538 (PMC11054217; doi:10.3390/pharmaceutics16040538)
Supplement: Supplementary file 1 [file pharmaceutics-16-00538-s001.zip › pharmaceutics-2920738-supplementary.pdf]

Supporting information

# In situ preparation of tannic acid-modified poly(*N*-isopropylacrylamide) hydrogel coatings for boosting cell response

Jufei Xu,[†,1,2] Xiangzhe Liu,[†,2,3] Pengpeng Liang,[4] and Hailong Yuan[*,1], Tianyou Yang[*,2]

[1] *Department of Pharmacy, Air Force Medical Center, PLA, Air Force Medical University, Beijing 100142, China*

[2] *School of Life Science and Technology, Henan Institute of Science and Technology, Xinxiang 453003, China*

[3] *University of Chinese Academy of Sciences, Beijing 100049, China*

[4] *School of Materials Science and Engineering, Zhengzhou University, Zhengzhou 450001, China*

*Corresponding author: E-mail address: yhlpharm@126.com (Hailong Yuan) (Hailong Yuan).

**Synthesis of hydroxyl-terminated poly(*N*-isopropylacrylamide) (PNIPAAm-OH)**

The radical telomerization of *N*-isopropylacrylamide (IPAAm) monomer using 2-mercaptoethanol (2-ME) as a chain transfer agent was used to prepare PNIPAAm-OH [1,2]. The synthesis mechanism of PNIPAAm-OH was shown in Scheme S1a. Briefly, IPAAm (5.0 g) and 2-ME (Aladdin, China) (0.3 g) and 2, 2′-Azobis (2-methylpropionitrile) (AIBN, 0.1 g) were dissolved in 50 ml of toluene. The mixture was stirred vigorously and degassed via aerating nitrogen gas for 40 min. The radical telomerization was carried out at 80°C for 4 h. After reaction, the resultant solution was filtered with 10 kDa membrane to ensure removing residual reagent. The collected compound was dried under vacuum and then further purified by repeated precipitation in diethyl ether from toluene.

X-ray photoelectron spectroscopy (XPS, Thermo Fisher Scientific Inc, USA) was carried out to confirm the successful preparation of PNIPAAm-OH. As depicted in Figure S1, a new XPS peak located at 162.1 eV compared with that of the pure PNIPAAm, which represents the $S_{2p}$. This indicates 2-ME may be introduced into PNIPAAm. Furthermore, the $S_{2p}$ was fitted with only one peak, denoting the C-S-C at 164.3 eV. The findings imply that the 2-ME was chemically bonded to the one end of PNIPAAm as the designed method in Scheme S1 a.

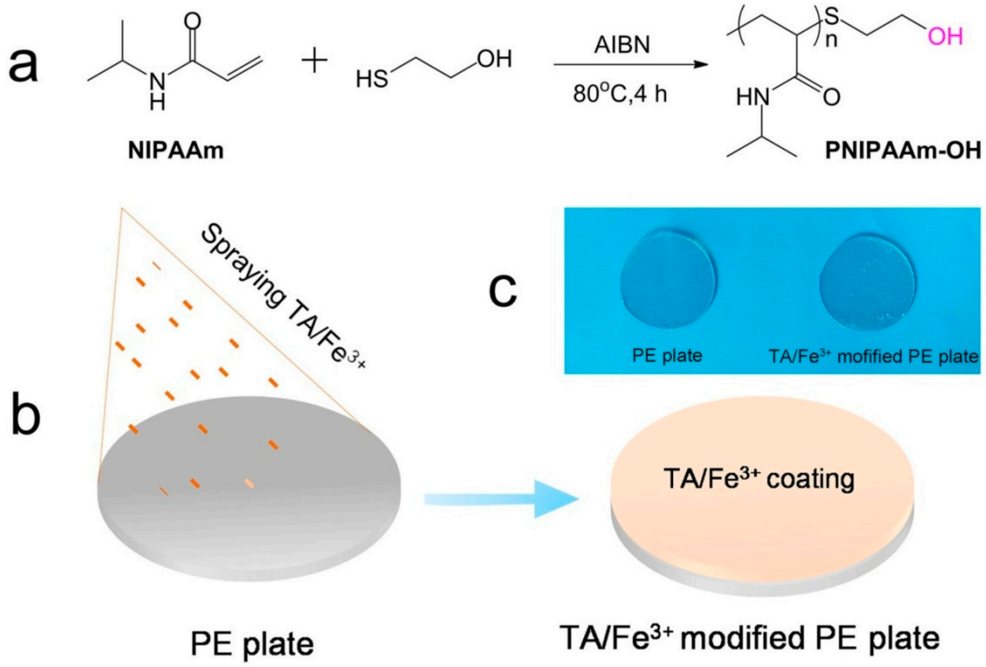


**Scheme S1** Preparation route and mechanism of PNIPAAm-OH (a), the preparation of TA/$Fe^{3+}$ modified PE plate (b) and their optical images (c).

**TA/$Fe^{3+}$ modified the surface of PE plate**

TA like the Polydopamine pocess the capability to adhere universal materials. In addtion, the complex of TA with iron ion ($Fe^{3+}$) is stable When pH value is above 7.0 [3]. So the TA/$Fe^{3+}$ was utilized to modify the inert surface of PE in order to stably fix the hydrogels coatings formed in the next stage. TA and $FeCl_3 \cdot 6H_2O$ (Aladdin, Chain) was diluted/disolved by DI water to get the concentrations of both 0.42 mg/mL for TA and 0.11 mg/mL for $FeCl_3$ following by adjusting pH to 7.0 using NaOH (Aladdin, Chain). The resultant TA/$Fe^{3+}$ solution was sputter-coated rapidly on the surfacse of PE plates. Then, the PE paltes were got through washing with DI water three times to remove unbound TA and $FeCl_3 \cdot 6H_2O$ and dried-freezing. The coatings preparation process was illustrated by Scheme S1 b. In addition, Scheme S1 c was presented the optical images of PE plates and TA/$Fe^{3+}$modified PE plate.

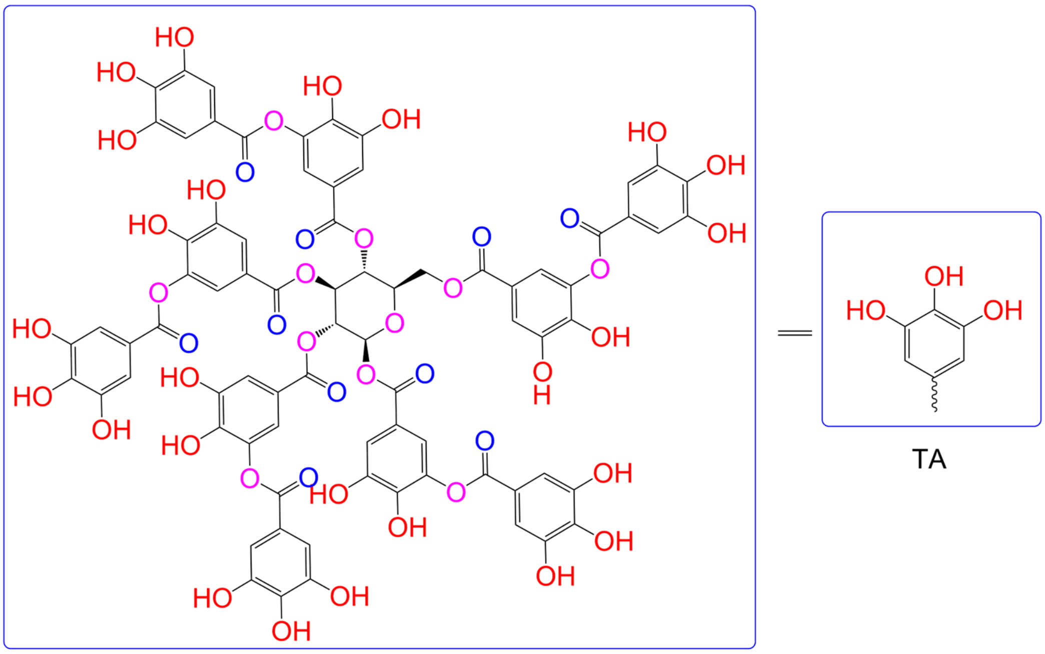

TA    TA

**Scheme S2** Chemical structure of TA. The two structures were regarded as be equal and the right one was used to represent the left in the Scheme 1.

Note: TA of PNIPAAm-APTES-TA in Scheme 1a only represents the chemical bonding TA. TA in PNIPAAm-APTES-TA in Scheme 1b and c refers to all TAs incorporated into coatings, including not only the chemical bonding TA but also non-chemical bonding TA.

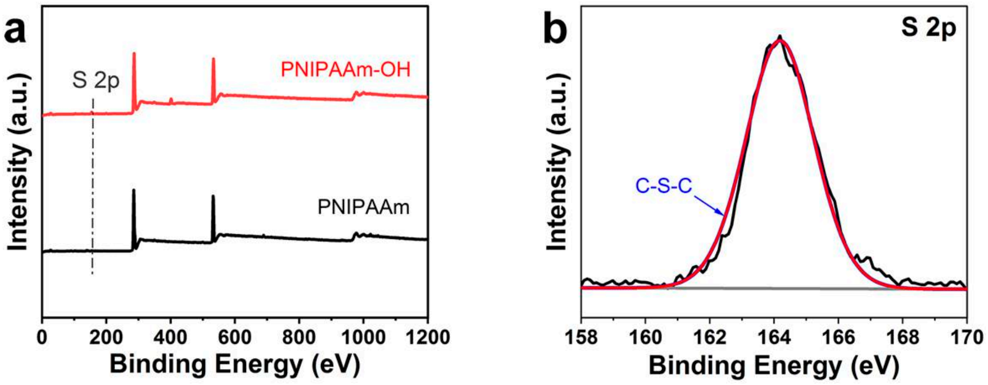


**Figure S1** The full spectra of XPS for PNIPNNm and PNIPAAm-OH (a) and the fitting spectra of $S_{2p}$ for PNIPAAm-OH (b).

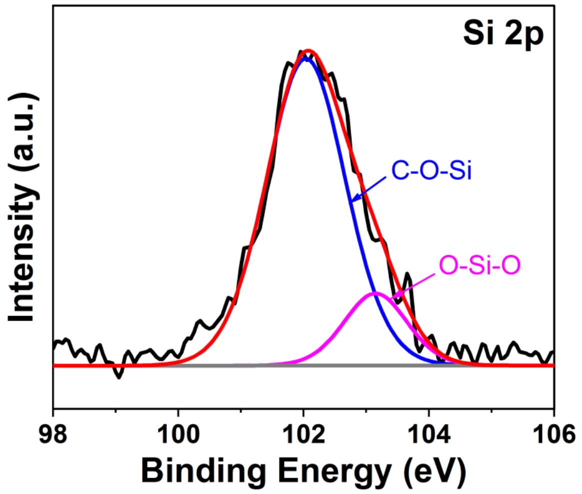


**Figure S2** The fitting spectra of $Si_{2p}$ for PNIPAAm-APEST.

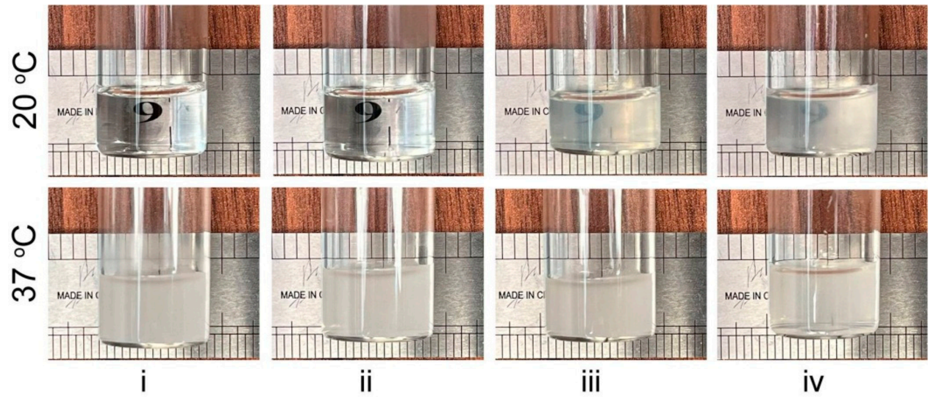


**Figure S3** Photograph of the coatings in PBS at 20 and 37°C. i: $PNIPAAm_{2000}$-APTES-TA, ii: $PNIPAAm_{1300}$-APTES-TA, iii: $PNIPAAm_{860}$-APTES-TA and iv: $PNIPAAm_{500}$-APTES-TA.

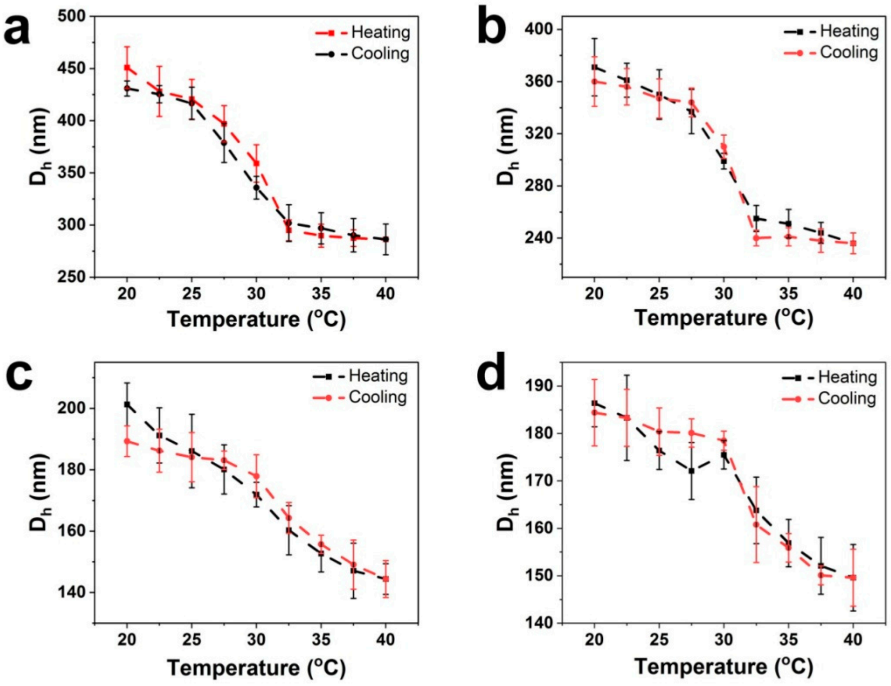


**Figure S4** Alternation of hydrodynamic radius with temperature: $PNIPAAm_{2000}$-APTES-TA (a),

PNIPAAm$_{1300}$-APTES-TA (b), PNIPAAm$_{860}$-APTES-TA (c) and PNIPAAm$_{500}$-APTES-TA (d).

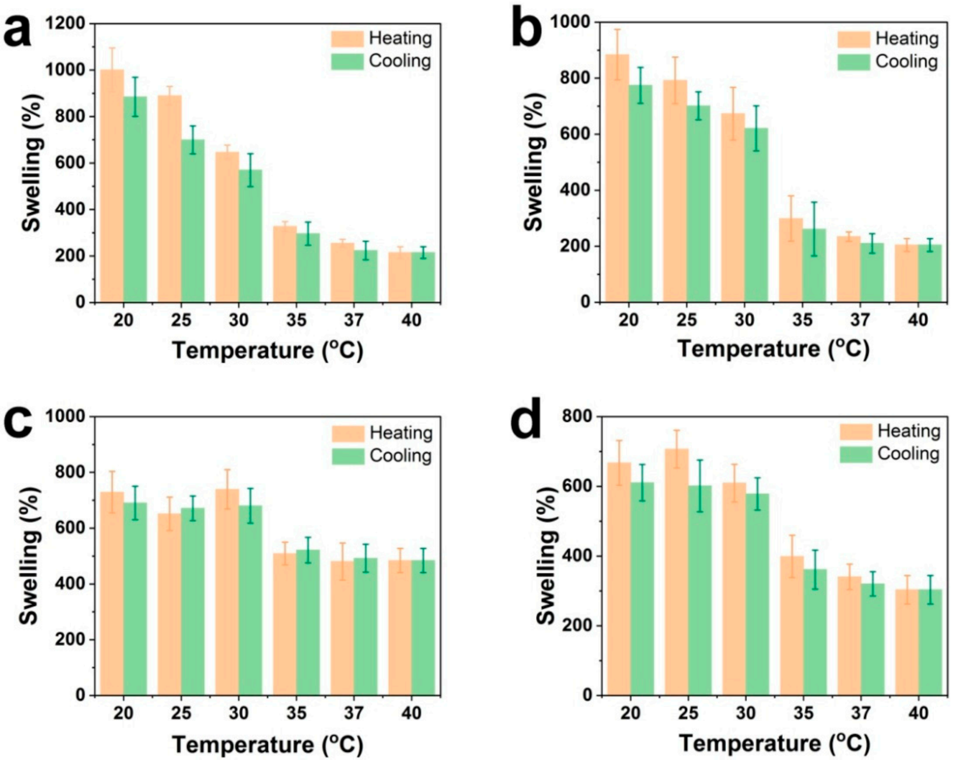


**Figure S5** Swelling rates of various coating with temperature: PNIPAAm$_{2000}$-APTES (a), PNIPAAm$_{1300}$-APTES (b), PNIPAAm$_{860}$-APTES (c) and PNIPAAm$_{500}$-APTES (d).

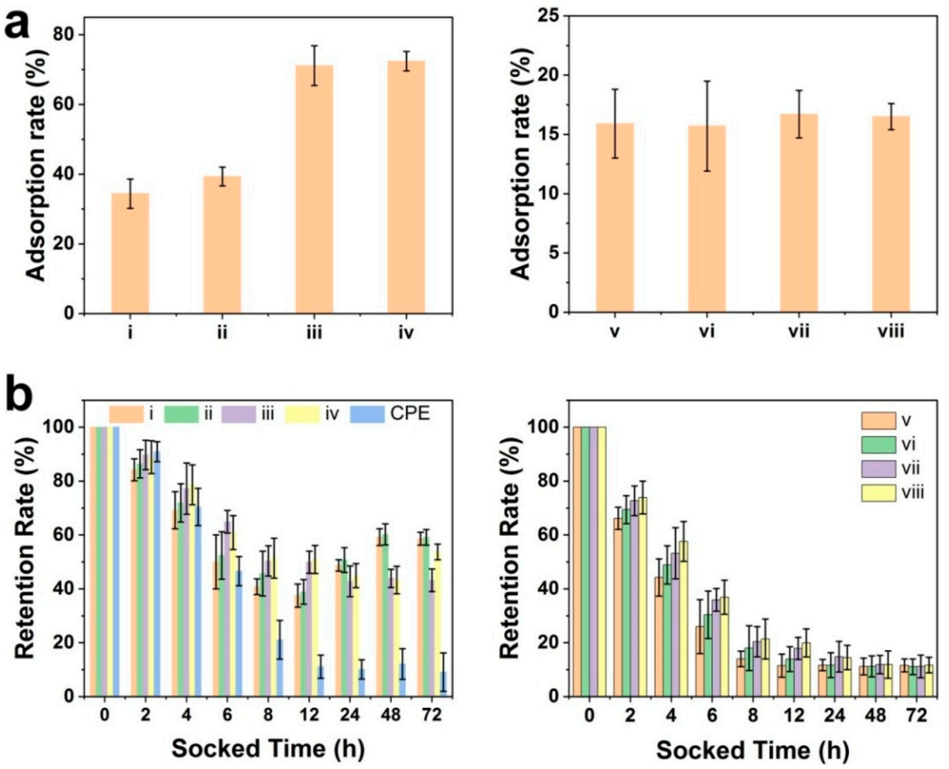


**Figure S6** Adsorption rate (a) and retention rate (b) of diverse coatings: PNIPAAm$_{2000}$-APTES-TA

(i), $PNIPAAm_{1300}$-APTES-TA (ii), $PNIPAAm_{860}$-APTES-TA (iii), $PNIPAAm_{500}$-APTES-TA (iv), $PNIPAAm_{2000}$-APTES (v), $PNIPAAm_{1300}$-APTES (vi), $PNIPAAm_{860}$-APTES (vii) and $PNIPAAm_{500}$-APTES (viii)

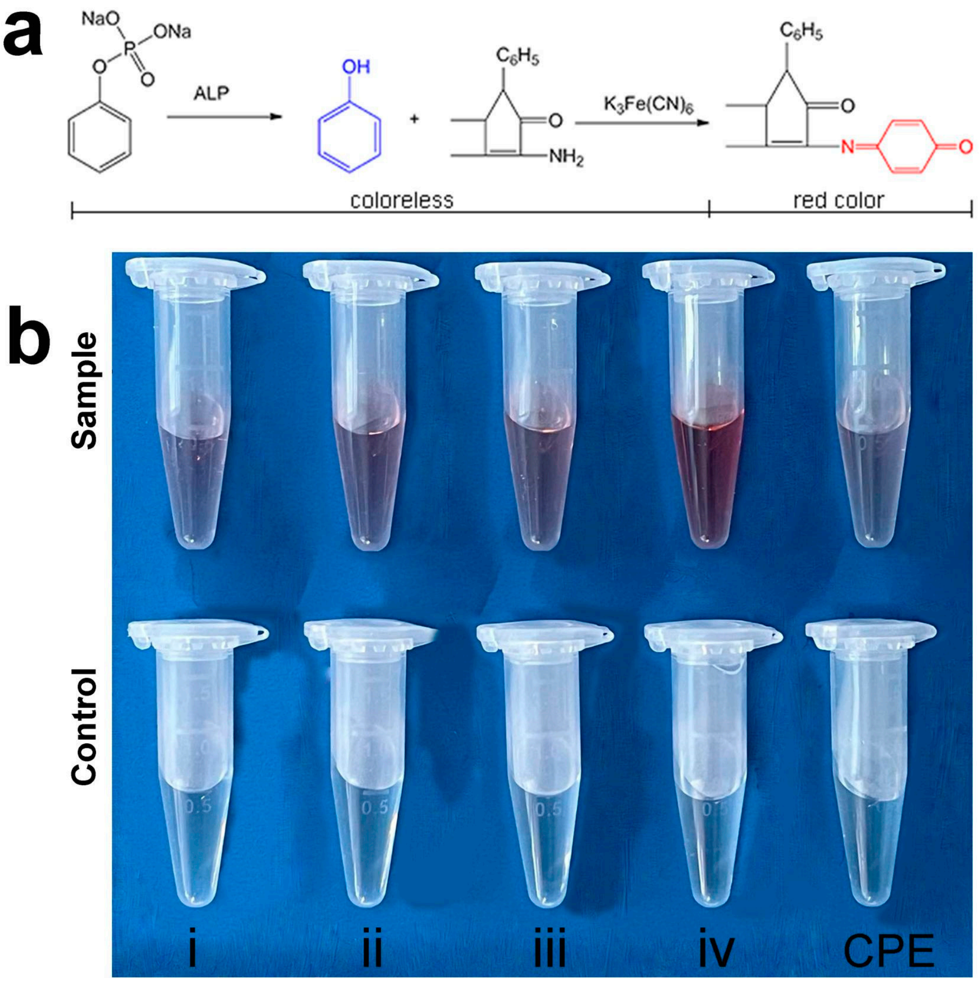


**Figure S7** The mechanism of ALP bioactivity detection (a) and optical images of various ALP reagent treated by ALP-adsorbed coatings or non-ALP coatings at 37°C for 30 min (b). $PNIPAAm_{2000}$-APTES-TA (i), $PNIPAAm_{1300}$-APTES-TA (ii), $PNIPAAm_{860}$-APTES-TA (iii) and $PNIPAAm_{500}$-APTES-TA (iv).

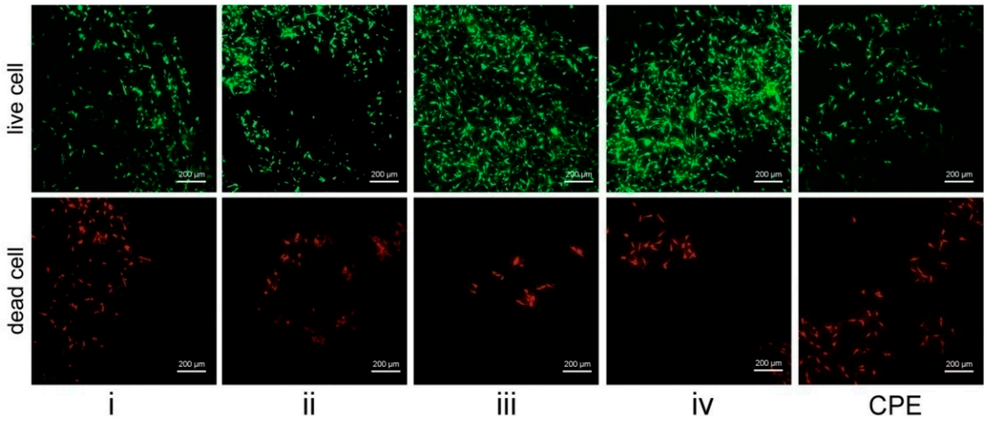


**Figure S8** The dead/live staining CLSM images of L929 cells. $PNIPAAm_{2000}$-APTES-TA (i),

PNIPAAm$_{1300}$-APTES-TA (ii), PNIPAAm$_{860}$-APTES-TA (iii) and PNIPAAm$_{500}$-APTES-TA (iv).

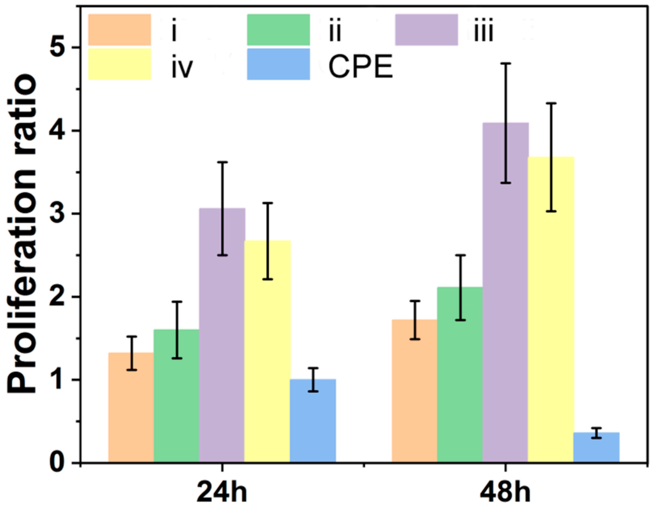


**Figure S9** Proliferation ratio of L929 cells on various surfaces. The cell number on CPE at 24 h acted as control. PNIPAAm$_{2000}$-APTES-TA (i), PNIPAAm$_{1300}$-APTES-TA (ii), PNIPAAm$_{860}$-APTES-TA (iii) and PNIPAAm$_{500}$-APTES-TA (iv).

**Table S1** The preparation condition for a variety of PNIPAAm-APTES

| Sample code | Abbreviation | Solution | |
|---|---|---|---|
| | | PNIPAAm-OH (mg) | APTES (μL) |
| V | PNIPAAm$_{2000}$-APTES | 2000 | 100 |
| VI | PNIPAAm$_{1300}$-APTES | 1300 | 100 |
| VII | PNIPAAm$_{860}$-APTES | 860 | 100 |
| VIII | PNIPAAm$_{500}$-APTES | 500 | 100 |

**Table S2** Content of various silicon-containing groups in PNIPAAm-APTES.

| Sample code | O-Si-O (%) | C-O-Si (%) |
|---|---|---|
| PNIPAAm$_{2000}$-APTES | 5.9 | 94.1 |
| PNIPAAm$_{1300}$-APTES-TA | 18.0 | 82.0 |
| PNIPAAm$_{860}$-APTES-TA | 23.8 | 76.2 |
| PNIPAAm$_{500}$-APTES-TA | 31.0 | 69.0 |

**Table S3** Chemical component and content of surfaces of different PNIPAAm-APTES-TA

| Sample code | Elemental composition (At%) | | | | |
|---|---|---|---|---|---|
| | C (%) | O (%) | N (%) | S (%) | Si (%) |
| I | 73.64 | 16.41 | 6.75 | 2.64 | 0.56 |
| II | 71.90 | 17.62 | 7.06 | 2.47 | 0.95 |
| III | 64.97 | 24.05 | 7.33 | 2.22 | 1.47 |
| IV | 63.83 | 24.23 | 7.71 | 2.16 | 2.07 |
| PE | 97.08 | 2.92 | - | - | - |

## References


1. Choi, C.; Chae, S. Y.; Nah, J.W., Thermosensitive poly(*N*-isopropylacrylamide)-b-poly(ε-caprolactone) nanoparticles for efficient drug delivery system. Polymer 2006**,** 47, 4571-4580.
2. Tamura, A.; Uchida, K.; Yajima, H., Reversible temperature-dependent dispersion-aggregation transition of poly(*N*-isopropylacrylamide)-60 Fullerene conjugates, Chem. Lett. 2006, 35, 282-283.
3. Ejima, H.;. Richardson, J.J.; Liang, K.; Best, J.P.; Koeverden, M.P.; Such, G.K.: Cui, J.W.; Caruso, F., One-step assembly of coordination complexes for versatile film and particle engineering, Science 2013, 341, 154-157.
